# Adaptive radiotherapy for muscle invasive bladder cancer: a retrospective audit of two bladder filling protocols

**DOI:** 10.1186/s13014-024-02484-9

**Published:** 2024-07-19

**Authors:** Diana Nohemi Briceño Guel, Nicola Laverick, Linda MacLaren, Nicholas MacLeod, Martin Glegg, Gillian Lamb, Peter Houston, Ross Carruthers, Laura Grocutt, Ronan M. Valentine

**Affiliations:** 1 College of Medical, Veterinary and Life Sciences, University of Glasgow, Glasgow, G12 8QQ UK; 2 Radiotherapy Physics, Department of Clinical Physics and Bioengineering, Beatson West of Scotland Cancer Centre, NHS Greater Glasgow and Clyde, Glasgow, G12 0YN UK; 3 Beatson West of Scotland Cancer Centre, NHS Greater Glasgow and Clyde, Glasgow, G12 0YN UK; 4 CRUK RadNet Glasgow, University of Glasgow, Glasgow, G61 1QH UK

**Keywords:** Muscle-invasive bladder cancer, Bladder volumes, Adaptive radiotherapy, Drinking protocols

## Abstract

**Background:**

Radical radiotherapy for muscle-invasive bladder cancer (MIBC) is challenging due to large variations in bladder shape, size and volume during treatment, with drinking protocols often employed to mitigate geometric uncertainties. Utilising adaptive radiotherapy together with CBCT imaging to select a treatment plan that best fits the bladder target and reduce normal tissue irradiation is an attractive option to compensate for anatomical changes. The aim of this retrospective study was to compare a bladder empty (BE) protocol to a bladder filling (BF) protocol with regards to variations in target volumes, plan of the day (PoD) selection and plan dosimetry throughout treatment.

**Methods:**

Forty patients were included in the study; twenty were treated with a BE protocol and twenty with a BF protocol to a total prescribed dose of 55 Gy in 20 fractions. Small, medium and large bladder plans were generated using three different CTV to PTV margins. Bladder (CTV) volumes were delineated on planning CTs and online pre-treatment CBCTs. Differences in CTV volumes throughout treatment, plan selection, PTV volumes and resulting dose metrics were compared for both protocols.

**Results:**

Mean bladder volume differed significantly on both the planning CTs and online pre-treatment CBCTs between the protocols (*p* < 0.05). Significant differences in bladder volumes were observed between the planning CT and pre-treatment CBCTs for BF (*p* < 0.05) but not for BE (*p* = 0.11). Both protocols saw a significant decrease in bladder volume between first and final treatment fractions (*p* < 0.05). Medium plans were preferentially selected for BE whilst when using the BF protocol the small plan was chosen most frequently. With no significant change to PTV coverage between the protocols, the volume of body receiving 25.0–45.8 Gy was found to be significantly smaller for BE patients (*p* < 0.05).

**Conclusions:**

This work provides evidence in favour of a BE protocol compared to a BF protocol for radical radiotherapy for MIBC. The smaller treatment volumes observed in the BE protocol led to reduced OAR and total body doses and were also observed to be more consistent throughout the treatment course. These results highlight improvements in dosimetry for patients who undergo a BE protocol for MIBC.

## Background

Bladder cancer is the tenth most common cancer diagnosis worldwide, with approximately 550,000 new cases and more than 200,000 deaths occurring each year [1, 2]. Radical cystectomy (RC) is considered the standard of care treatment for muscle-invasive bladder cancer (MIBC) [3]. Radiotherapy (RT) delivered with or without radiosensitisation over 4–7 weeks is a well-established radical treatment regimen for MIBC [4]. While studies comparing RC to RT have shown comparable overall survival outcomes in appropriately selected patients, RT has the potential to safeguard organ function [5, 6]. A treatment paradigm involving four weeks of daily hypofractionated RT, delivering a total dose of 55 Gy in 20 fractions is widely accepted as standard of care for MIBC in the UK [4].

Radical RT for MIBC is technically challenging due to the geometric uncertainty of the bladder target throughout treatment. Variations in bladder volume, shape and position together with the influence of bladder filling and movement of nearby organs can lead to large inter- and intra- fractional variations. Large population-based margins have been employed to overcome the uncertainty associated with such a mobile target, however, this results in unnecessary normal tissue irradiation and increased toxicity coupled with the risk of geometric misses [7–9].

Adaptive radiotherapy (ART) refers to the modification of a RT treatment plan to account for changes in patients’ anatomy, e.g., tumour shape and bladder filling. The overall aim of ART is to deliver the prescribed dose to the target with high precision and accuracy while reducing margins and limiting normal tissue irradiation [10, 11]. There are a number of different approaches that can be taken to deliver ART. As defined by a Royal College of Radiologists (RCR) guidance document, these can fall into four categories; Reactive ART, Scheduled ART, Proactive ART and Real-time ART [12]. The latter two approaches can essentially be classified as online ART (oART) whereby an appropriate treatment plan is either chosen from a library of plans (Proactive ART) or a new plan is generated at the time of treatment, after daily pre-treatment imaging has been acquired (Real time ART). Optimisation of target coverage has been realised through Image Guided Radiotherapy (IGRT) and the use of soft tissue imaging, such as Cone Beam Computed Tomography (CBCT), which offers assessments of inter- and intra- fractional variations before and during treatment delivery. CBCT imaging has facilitated the oART delivery approach, which is becoming more widely available and is desirable given its potential to generate individualised treatment strategies. For radical oART for MIBC the strategy involving a library of plans for each patient has been shown to be clinically feasible and generally well-tolerated [13, 14] with three plans being commonly recommended due to offsets between resources, time and the overall benefits offered to patients [15]. This library of plans strategy consists of a “plan of the day” (PoD) delivery approach combined with daily CBCT imaging to select a treatment plan that best fits the bladder target and minimises normal tissue irradiation. Using the PoD approach aims to account for bladder filling, volume, shape and position at each fraction and has been shown to reduce normal tissue irradiation compared to a single plan delivery approach [16–20].

Since participating in the RAIDER clinical trial our centre has adopted the PoD technique [21]. Prior to December 2021, standard clinical practice involved generating a library of plans (small, medium and large) with a bladder filling (BF) drinking protocol as described in the RAIDER clinical trial protocol. Subsequent to the RAIDER clinical trial there has been increasing interest in the use of BE protocols, but there is no evidence as of yet to support adoption of BE protocols across all centres. While the majority of radiotherapy centres employ a BE protocol [14–19, 22, 23], others have elected to use a BF protocol [24–26].

Previous studies have suggested that an empty protocol offers improved bladder volume reproducibility and a reduction of the irradiated volume [16–19]. An empty protocol presents the advantage of patients not having to drink and retain water, which offers a more comfortable experience. Conversely a BE protocol may be more challenging to accomplish for patients as they progress through treatment due to swelling and incomplete bladder emptying related to toxicity [27]. Finally, filling protocols may afford greater sparing of organs at risk (OARs) [24–26]. There is a relative paucity of recently published data reporting on volumetric differences coupled with dosimetric differences between empty and filling PoD protocols for MIBC.

The aim of this retrospective audit was to assess the impact of these two different drinking protocols, on a variety of oART treatment parameters for MIBC, including bladder volumes recorded on planning CT (pCTs) and CBCTs, bladder filling rates, plan selection, planning target volume (PTV) volumes, OAR doses and normal tissue irradiation volumes.

## Methods

### Selection of patients

40 patients who had been treated with radical radiotherapy for MIBC (staged at T2-T4aN0M0) between 2021 and 2023 were selected. Both protocol groups, i.e., bladder filling (BF) and bladder empty (BE), consisted of 20 patients each and were chosen at random from patient lists associated with calendar years 2021 (BF) and 2022/2023 (BE). Informed consent was obtained for each patient with permission requested to use their imaging data for additional research beyond clinical treatment. All patients were prescribed 55 Gy in 20 fractions and received oART coupled with VMAT and IGRT. Patients having a PTV that extended outside the external contour, significant artefacts in the CBCT images, or an additional prostate cancer prescription were excluded from the study. The BE protocol group was made up of 3 female and 17 male patients with ages ranging from 56 to 84 (mean 73) years. Two out of 20 patients received 18 fractions. In one case the early completion was attributed to chemotherapy toxicities, the reason in the other case is unknown. All other patients received 20 fractions. The BF protocol consisted of 5 female and 15 male patients and an age range from 55 to 85 (mean 75) years. All 20 patients received the prescribed 20 fractions.

### CT simulation

As mentioned previously, the BF protocol previously adopted by the department was consistent with the RAIDER clinical trial [21]. Patients were advised to void their bladder, drink 350 ml of water then wait 30 min before their first planning CT (pCT) scan with a second pCT scan acquired a further 30 min later. Current practice employs the BE protocol, where patients are instructed to void their bladder immediately before their first pCT then wait 30 min for a second pCT. All patients were scanned supine with arms across the chest and immobilised using a headrest and prostep immobilisation device. OARs delineated comprised the rectum, bowel loops, bowel cavity and femoral heads (left and right). The CTV was defined as the entire bladder volume. Anisotropic expansions of clinician defined bladder volumes, i.e., CTV to PTV consistent with the RAIDER trial were generated as shown in Table 1. PTV_Small and PTV_Medium are both grown from the CTV delineated on the first planning CT (acquired at 0 min for the BE protocol and 30 min for the BF protocol). The process for PTV_Large is dependent upon the change in bladder size between the two planning CT scans. If the volume difference in the CTV is less than 50 ml, PTV_Large is also grown from the CTV on the first pCT scan. If the difference is greater than 50 ml, it is grown from the CTV on the second pCT scan following a different margin protocol. All volumes are propagated to the first pCT scan for plan generation.

**Table 1:** CTV to PTV expansion for adaptive bladder plans. Identical margins were used for both drinking protocols

CT data set	PTV	CTV to PTV expansion (cm)				
		Laterally	Anteriorly	Posteriorly	Superiorly	Inferiorly
CT_0	PTV Small	0.5	0.5	0.5	0.5	0.5
CT_0	PTV Medium	0.5	1.5	1.0	1.5	0.5
If CT_30 – CT_0 < 50mls then						
CT_0	PTV Large0	0.8	2.0	1.2	2.5	0.8
If CT_30 – CT_0 > 50mls then						
CT_30	PTV Large30	0.5	1.5	1.0	1.5	0.5

### Radiotherapy technique

All plans in this study were generated using the Eclipse™ Treatment Planning System (TPS) (Varian Medical Systems). The algorithm was upgraded from Acuros v15.5 to v16.1 within the time window of this cohort. Re-calculation tests, however, showed no significant differences in calculated dose. In all cases, a dose calculation grid size of 2.5 mm was used. The VMAT technique was used for all plans utilising 2 full arcs with jaw tracking applied, the collimator rotated to 30 degrees for clockwise arc rotation and 330 degrees for counter clockwise arc rotation and beam energy of 6MV. Plans were produced for Truebeam linear accelerators (Varian Medical Systems) fitted with both Millennium and HD multi-leaf collimators. Planning constraints specified for the OARs are in accordance with the RAIDER clinical trial while the PTV planning constraints were as follows; D99% > 90%, D95% > 95%, D5% < 105% and D2% < 107%.

### Treatment delivery

For treatment delivery all patients underwent the same bladder preparation as they received at the time of their CT simulation. CBCT imaging was undertaken immediately prior to each treatment fraction allowing for adjustments to the treatment position and ensuring optimal coverage of the target. As per routine clinical practice, post-treatment CBCT scans were acquired at fraction one and once-weekly to monitor intra-fraction bladder filling. The CBCT – irrespective of bladder drinking protocol – was assessed in all planes to select the appropriate plan to cover the CTV including a small margin to allow for intra-fraction bladder filling. The plan deemed most suitable and selected as plan of the day was one that offered optimal target coverage alongside minimal normal tissue irradiation, ensuring the CTV was fully encompassed in all directions by the smallest PTV possible. To ensure high concordance in plan selection, each CBCT assessment was carried out by two therapeutic radiographers who had completed a robust competency-based training package that was developed from the RAIDER clinical trial guidelines [21].

### Bladder volume analysis

For all patients, bladder outlining was performed on the pre-treatment CBCTs acquired at treatment fractions, 1, 2, 3, 6, 11, 16 and 20 (fraction 18 in two cases for the BE protocol), resulting in a total of 280 CBCTs analysed for this study. The chosen sample of seven fractions was deemed to be representative of any variation that may occur during the treatment course [28]. Bladder volumes as delineated by the prescribing clinician on the pCTs for both protocol groups were recorded. All contours outlined on the CBCTs were generated by an MSc student, which were subsequently checked by two physicists followed by a final review carried out by an experienced therapeutic radiographer.

Firstly, the bladder volumes outlined on the first and second pCTs were compared for both protocol groups and filling rates were estimated by dividing the volume increase between the first and second pCTs by the time between scans, i.e. 30 min. Next, bladder volumes outlined on each of the seven CBCTs were analysed for both groups. The difference between mean bladder volumes recorded at simulation (first pCT) and mean bladder volumes on treatment (CBCTs) for both groups were compared. Finally, mean bladder volumes for each of the seven fractions were compared both within- and between- protocol groups with particular attention given to differences recorded between the first and last fractions.

### Plan selection analysis

The number of times a particular plan was selected for treatment for each patient and the associated PTV volume were recorded. Mean “treated” PTV volumes were calculated for each patient depending on the number of varying plan sizes delivered over the course of treatment. Finally, for each patient it was determined which plan they received most frequently over the full course of their treatment.

### Dosimetric evaluation

OAR dose metrics for each treatment plan were retrieved from the Eclipse TPS. To calculate the OAR doses received by each patient, a “Plan Sum” consisting of the plans the patient received during their treatment course was generated to model the delivered treatment course. For example, a plan sum may have been comprised of 5 PTV_Large, 5 PTV_Medium and 10 PTV_Small plans. The plan sum is calculated and displayed on the first pCT. Differences in OAR doses between both groups were analysed. PTV statistics for both groups were also recorded and documented. Finally, the volume of specific isodose structures were compared in an effort to determine if total body doses delivered, in other words normal tissue irradiation, was consistently and statistically different between protocol groups. The isodose structures created were based on pertinent OAR dose constraint values [4], i.e., normal tissue volumes receiving specific dose levels such as V25 Gy, V37.50 Gy, V41.7 Gy, V45.8 Gy, and V54.2 Gy.

### Statistical analysis

Population means were compared among both protocol groups, assuming that the observations were independent from each other and that they came from a normal distribution. With the exception of the ANOVA test to compare bladder volume differences between the first and last fractions for both protocols, all statistical analyses between protocol groups were performed using a two-sample t-test. The level for statistical significance was set to *p* < 0.05. All boxplots, graphs and statistical tests were undertaken using Origin 2023b (OriginPro 2023b) (OriginLab Corporation). The standard deviation (SD) was used as systematic error for the means and the Standard Error of Mean (SEM) as the error for the mean difference.

## Results

### Analysis of bladder volumes

**Fig. 1:**
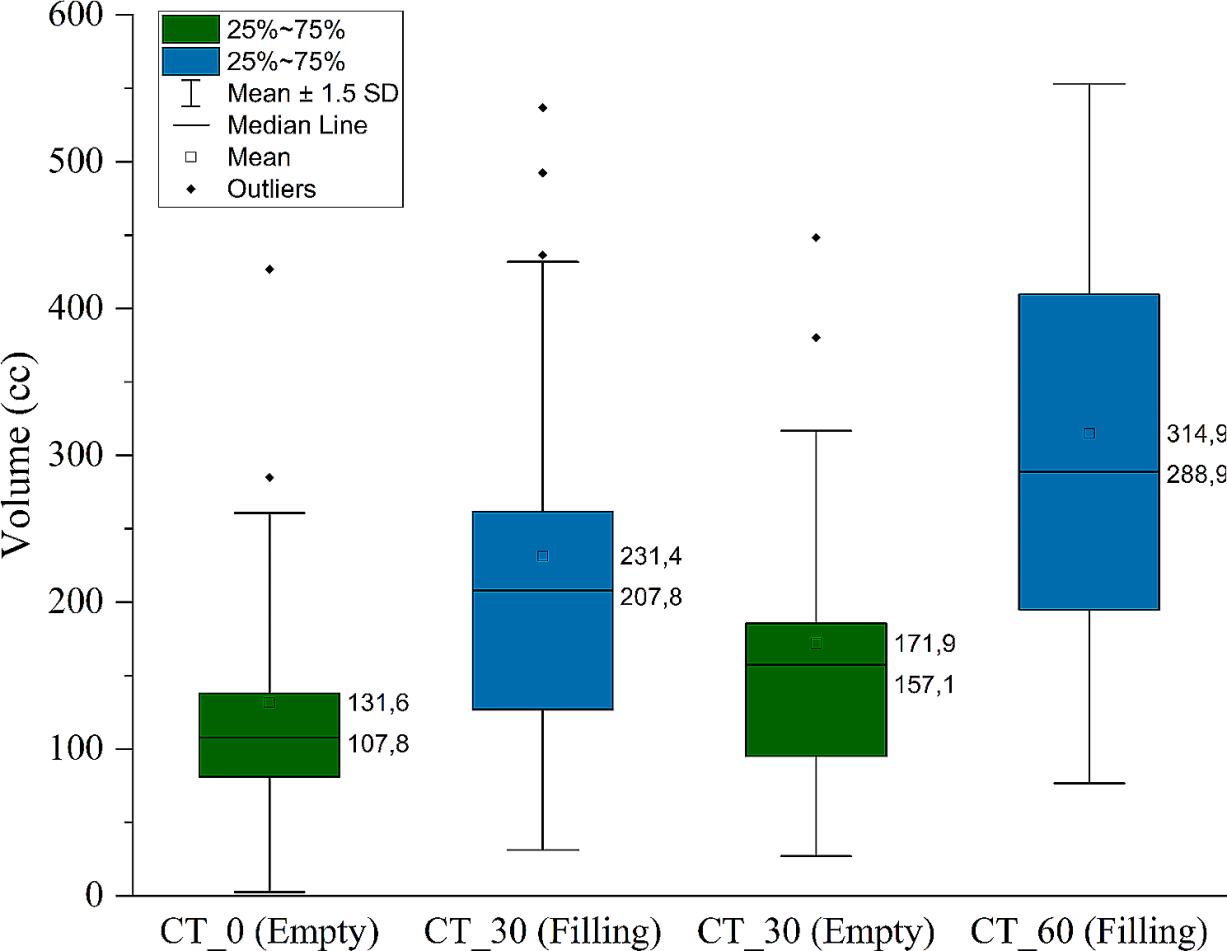
Bladder volumes on first and second pCTs for both BE and BF protocols. The volumes measured on the first pCT and second pCTs are significantly different between protocols (*p* < 0.05)

**Fig. 2:**
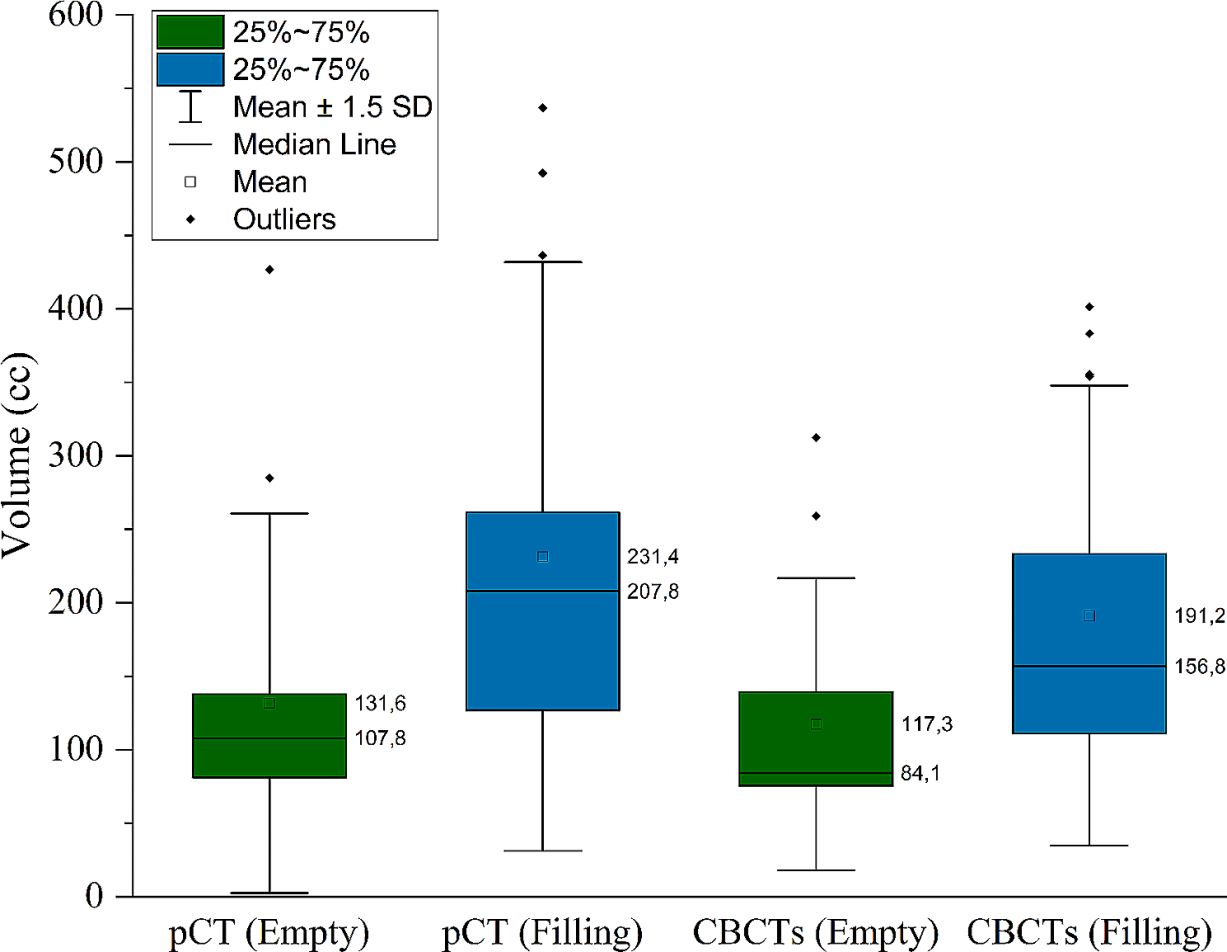
Comparison between bladder volumes on the first pCTs and on-treatment CBCTs for both BE and BF protocols. A significant difference was observed between the pCT volumes and CBCT volumes for the BF protocol (*p* < 0.05) but not for the BE protocol (*p* = 0.11)

**Fig. 3:**
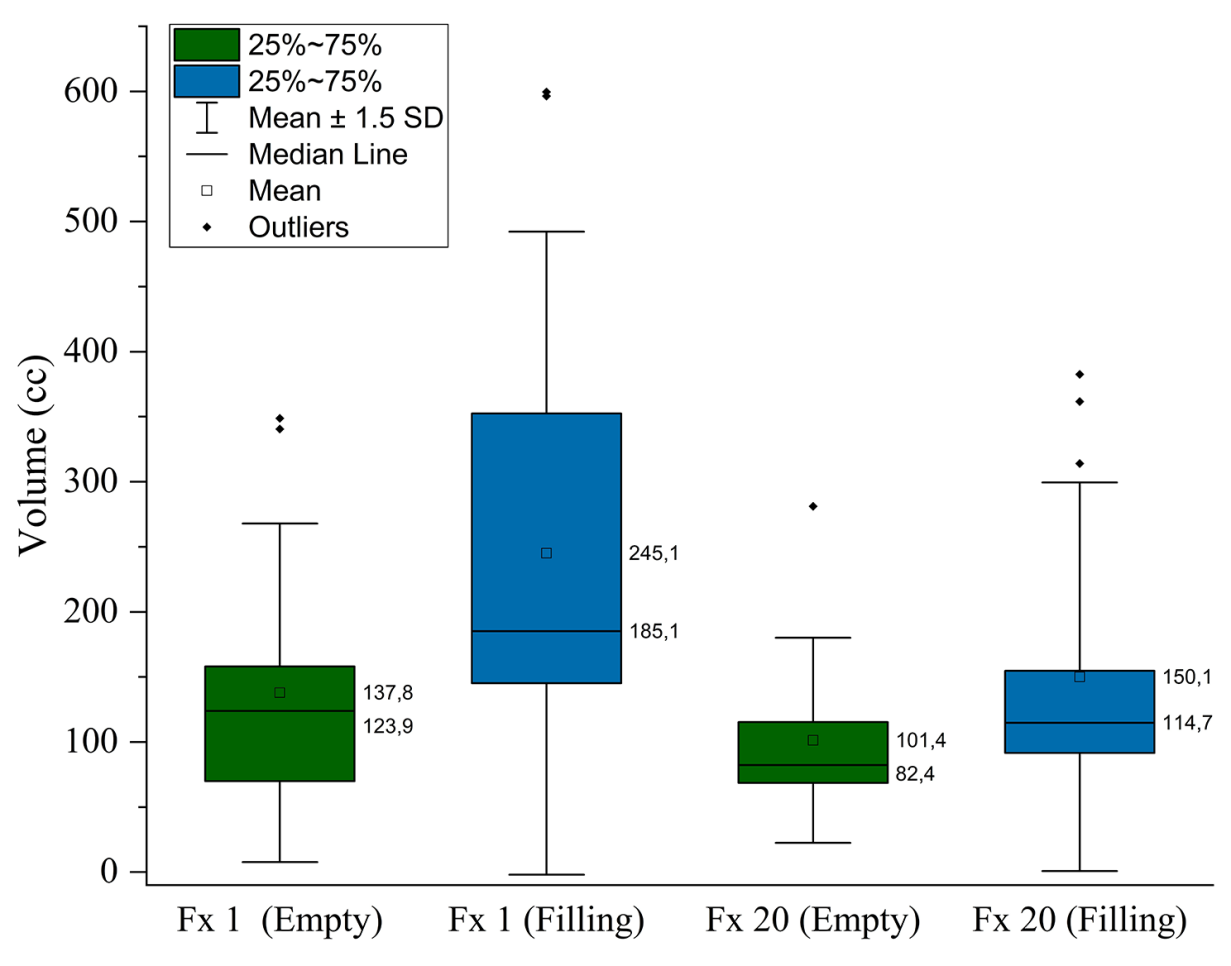
Bladder volumes at the first and last treatment fractions for BE and BF protocols. For both protocols, the difference in volume between first and last fractions is statistically significant (*p* < 0.05)

**Fig. 4:**
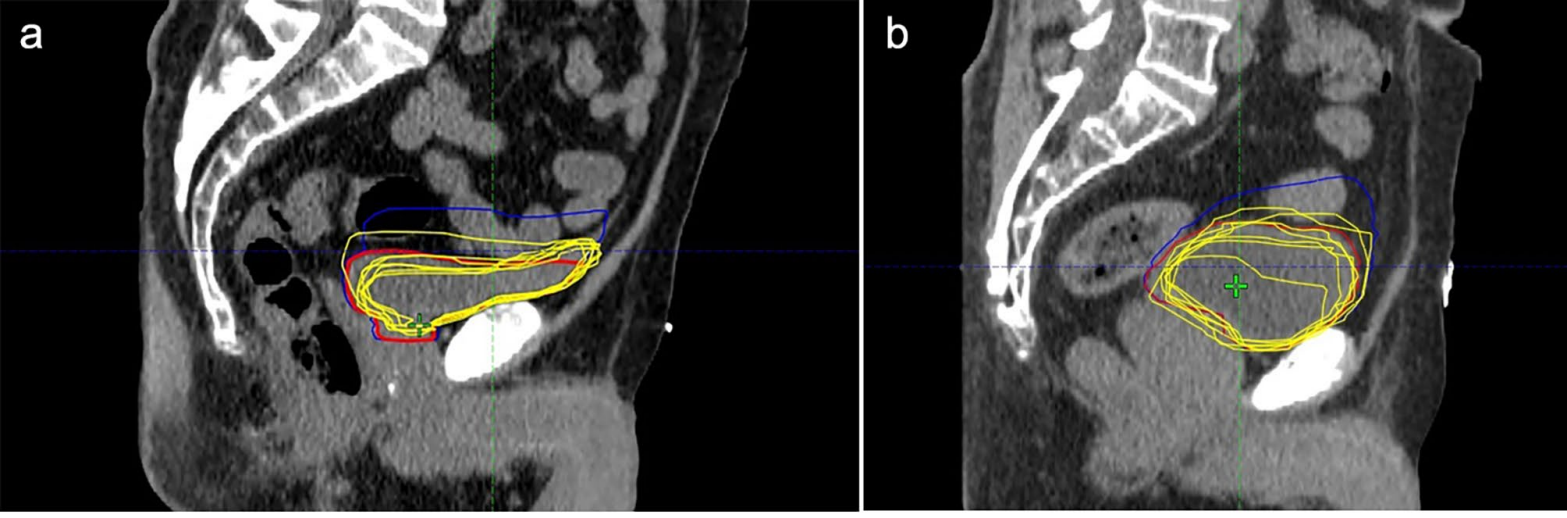
Sagittal CT slice for a patient from the BE (**4a**) and BF (**4b**) cohorts. Bladder volumes from treated fractions are shown in yellow, with the CTV from the first pCT in red and from the second pCT in blue. The larger variation in target size, shape and position with the BF protocol is evident

The pCT bladder volumes were compared between protocol groups as illustrated in Fig. 1. As expected, a statistical difference in mean bladder volume (cc) was found between the first pCTs (131.6 ± 19.3 cc [BE] vs. 231.4 ± 29.9 cc [BF]; *p* < 0.05) and second pCTs (171.9 ± 21.6 cc [BE] vs. 314 ± 35.5 cc [BF]; *p* < 0.05) between protocols which can be attributed to the difference in bladder filling. Also, a significantly larger mean filling rate (2.8 ± 0.4 cc/min) was observed for the BF protocol compared to the BE protocol (1.3 ± 0.2 cc/min) along with a greater variation between patients. Figure 2 illustrates a significantly reduced mean bladder volume recorded from CBCTs before treatment for the BE protocol compared to the BF protocol (117.3 ± 14.8 cc [BE] vs. 191.2 ± 23.3 cc [BF]; *p* < 0.05). While no significant difference in mean bladder volumes between the first pCT and on treatment CBCTs was observed for the BE protocol (Fig. 2; *p* = 0.11), there was a significant difference for the BF protocol (Fig. 2; *p* < 0.05), suggesting greater reproducibility between simulation and treatment for the BE protocol. On average, a gradual decrease in mean bladder volume was observed as the patients progressed through treatment from fraction 1 through to fraction 20. Figure 3 illustrates that for both bladder preparation protocols, significant decreases in volumes were noted particularly when comparing the first and final fractions (137.8 ± 19.4 cc vs. 101.4 ± 11.8 cc [BE; *p* < 0.05] and 245.1 ± 36.8 cc vs. 150.1 ± 22.3 cc [BF; *p* < 0.05]). Finally, the BF protocol exhibited a larger variation in bladder volume along with a greater decrease in volume from fraction 1 to fraction20. Figure 4 shows sagittal CT views of patients from the BE cohort (4 a) and BF cohort (4 b). For each patient, the bladder volumes delineated on the on-treatment CBCTs (yellow) are shown along with the CTVs from the first (red) and second (blue) pCTs. The increased variability observed for the BF protocol in comparison to the BE protocol is well illustrated.

### Plan selection

**Fig. 5:**
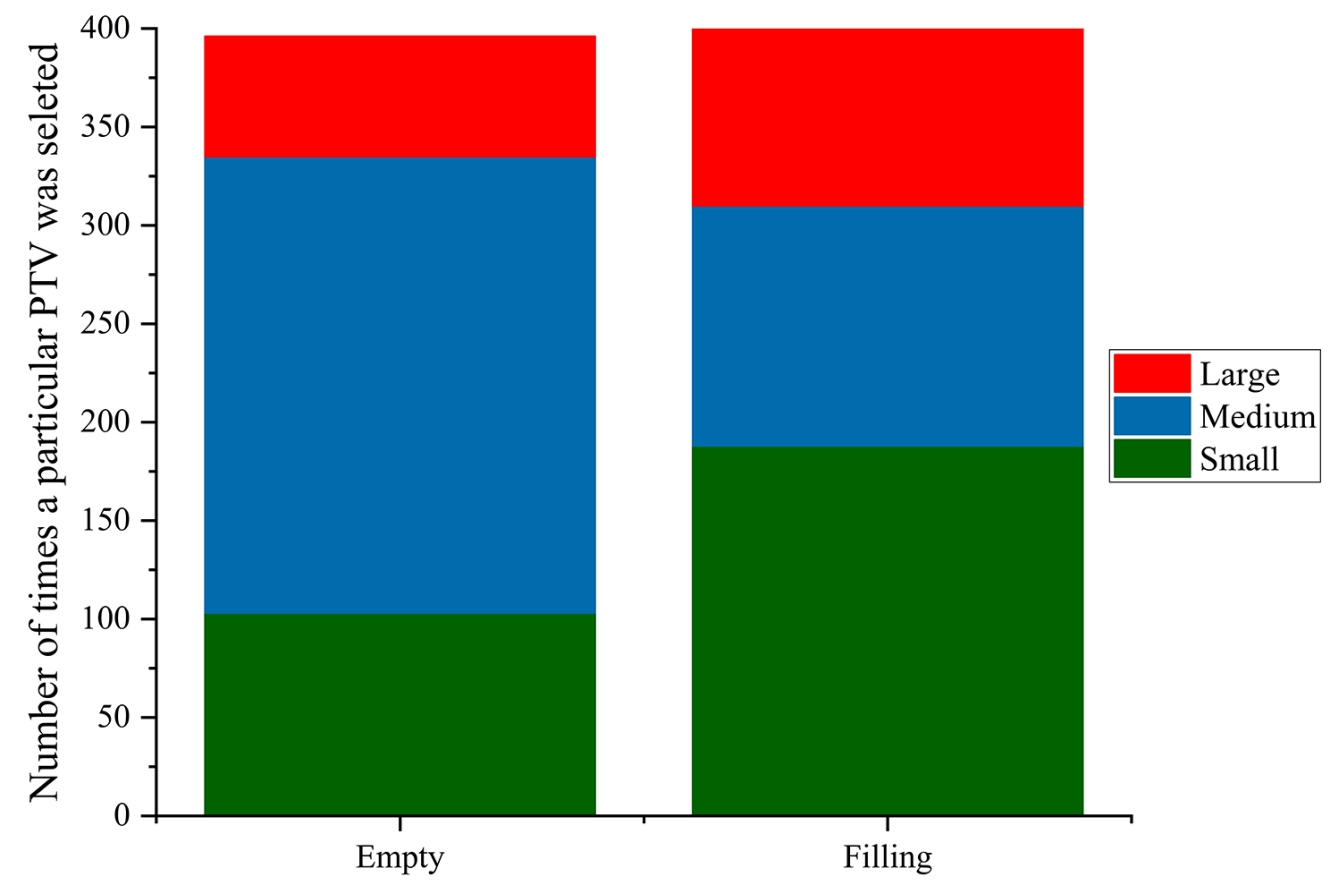
Choice of PoD over all patients (*n* = 40), over all treatment fractions. For the BE protocol (*n* = 20), the medium plans were most frequently chosen and for the BF protocol (*n* = 20) the small plans were most frequently chosen

**Fig. 6:**
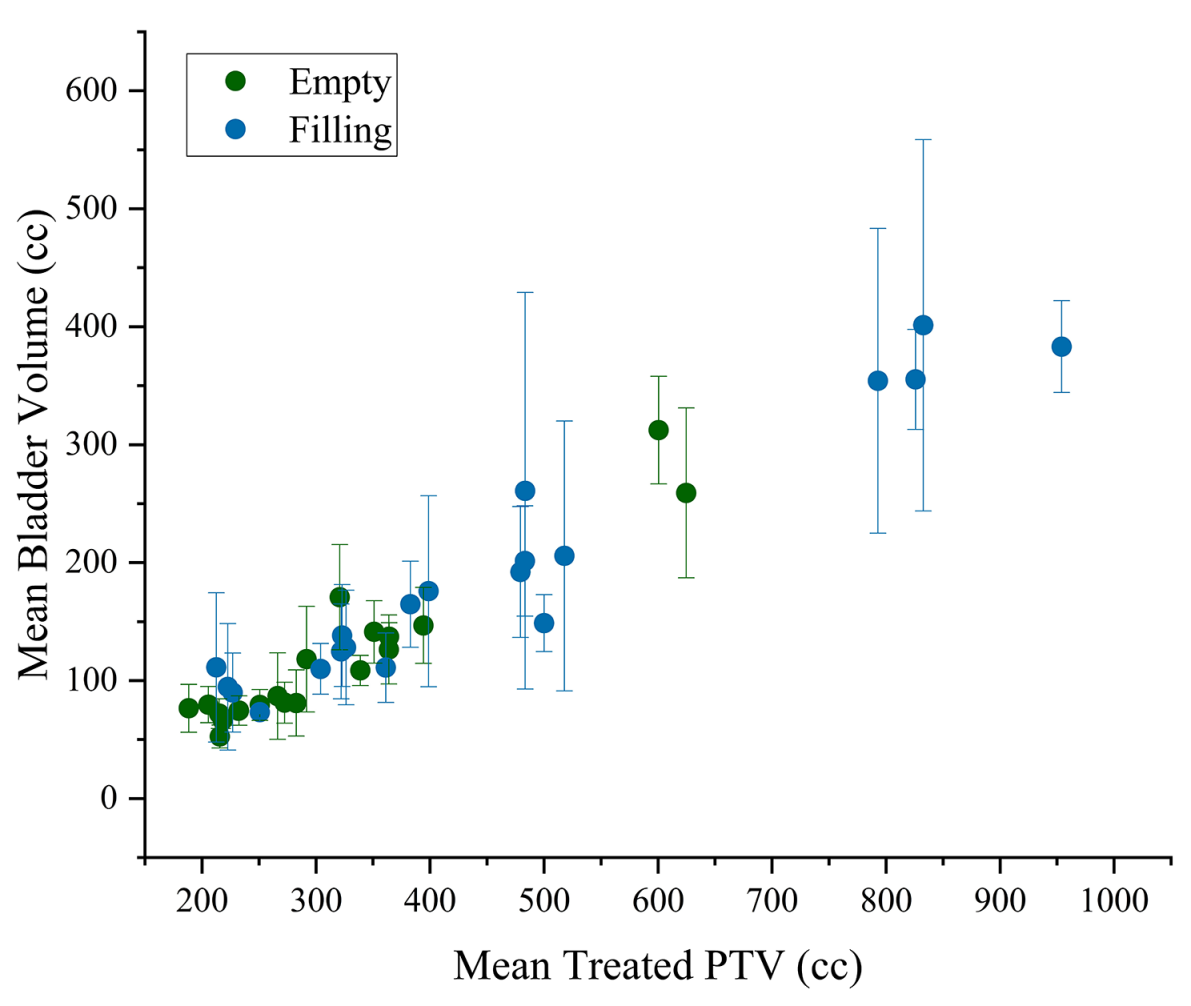
Mean bladder volume plotted against mean “treated” PTV volume for all patients in the study. As expected, there is a correlation between bladder volume on the CBCTs and the average size of the treated volume, with overall larger treated volumes seen for patients in the BF cohort

As described, the plan for each fraction is selected based solely upon the best choice for the patient’s anatomy on the day of treatment and can therefore vary over the course of treatment. For the BE protocol, 58.6% of all treatment plans selected over all patients and all treatment fractions were the medium plans while 26% were small and 15.4% large (Fig. 5). Conversely, Fig. 5 highlights that 47% of the total plans chosen for the BF protocol were small while medium plans were picked 30.5% of the time with large plans selected 22.5% of the time. Mean PTV volumes of medium plans in the BE group, 321.7 ± 135.8 cc (*n* = 20) were not significantly different from those of small plans in the BF group, 354.9 ± 177.7 cc (*n* = 20). Within the BE protocol group, medium plans were selected at least 10 times for 75% (15/20) of patients, while for the BF group, small plans were selected at least 10 times for 65% (13/20) of patients. We found a significant difference in mean treated PTV volumes between the BF protocol, 459.9 ± 223.5 cc and the BE protocol, 311.4 ± 119.5 cc (*p* < 0.05). As previously reported PTV volumes of selected plans correlated with daily bladder volumes on CBCT images [19]. In this study, the bladder volumes outlined on the seven pre-treatment CBCTs for all 20 patients within each protocol group were averaged and compared to their respective mean “treated” PTV volume recorded over the course of treatment. Figure 6 displays a similar correlation (Pearson’s R [BE = 0.95 and BF = 0.97]) illustrating that larger bladder volumes outlined on CBCTs results in the overall selection of relatively larger PTVs and associated treatment plans.

### Plan dosimetry

PTV coverage for each of the three oART plans created for both protocols met the mandatory dose-volume planning objectives. No discernible differences in dose statistics were found between protocols when comparing the small, medium and large PTVs. Dosimetric comparisons of the bowel loops and rectum were examined and showed that at each specified dose level for the bowel loops (Table 2) and rectum (Table 3) the volume of the OAR receiving that dose was consistently lower in the BE protocol group compared to the BF group. Furthermore, a significant difference in volume (*p* < 0.05) was found for the rectum at the V41.7 Gy and V50.0 Gy dose levels. In addition to evaluating PTV coverage and OAR doses, the volume of the body receiving specific dose levels was recorded as a surrogate of normal tissue irradiation. Table 4 highlights a significantly reduced volume (*p* < 0.05) of the body receiving low and medium doses (V25.0 Gy, V37.5 Gy, V41.7 Gy and V45.8 Gy) for the BE protocol strategy compared to the BF protocol strategy with the exception of the highest dose level, i.e., V54.2 Gy.

**Table 2:** Mean values (cc) with the SD as the systematic error for the bowel loops receiving specified dose levels. The mean difference is presented with the SME as the systematic error. The level for statistical significance was set to *p* < 0.05

Constraint	V37.5 Gy < 139 cc	V41.7 Gy < 127 cc	V45.8 Gy < 115 cc	V50.0 Gy < 98 cc	V54.2 Gy < 40 cc
	Mean ± SD (95% CI)	Mean ± SD (95% CI)	Mean ± SD (95% CI)	Mean ± SD (95% CI)	Mean ± SD (95% CI)
Empty Protocol	48.2 ± 30.3 (34.0–62.4)	40.6 ± 27 (28.0–53.2)	33.3 ± 23.7 (22.2–44.4)	22.7 ± 19.8 (13.4–31.9)	11.6 ± 12 (6.0–17.2)
Filling Protocol	71.7 ± 61.4 (43.0–100.5)	59.4 ± 54.3 (33.9–84.8)	47.8 ± 47.1 (25.8–69.9)	34.8 ± 37.6 (17.2–52.4)	12.5 ± 16.7 (4.7–20.3)
Mean difference	23.5 ± 15.3	18.8 ± 13.6	14.5 ± 11.8	12.1 ± 9.5	0.9 ± 4.6
*p*-value	0.13	0.18	0.23	0.21	0.85

**Table 3:** Mean values (%) with the SD as the systematic error for the rectum receiving specified dose levels. The mean difference is presented with the SME as the systematic error. The level for statistical significance was set to *p* < 0.05

Constraint	V 25.0 Gy < 80%	V 41.7 Gy < 60%	V 50.0 Gy < 50%	V 54.2 Gy < 30%	
	Mean ± SD (95% CI)	Mean ± SD (95% CI)	Mean ± SD (95% CI)	Mean ± SD (95% CI)	
Empty Protocol	39.5 ± 14.7 (32.6–46.4)	3.4 ± 4.3 (1.4–5.4)	1.1 ± 1.9 (0.2–2.0)	0.4 ± 0.8 (0.0–0.8)	
Filling Protocol	43.6 ± 20.7 (33.9–53.3)	10.5 ± 10.5 (5.6–15.4)	4.6 ± 5.2 (2.1–7)	0.9 ± 1.4 (0.3–1.6)	
Mean Difference	4.1 ± 5.7	7.1 ± 2.5	3.5 ± 1.2	0.6 ± 0.4	
*p*-value	0.48	0.01	0.01	0.13	

**Table 4:** Mean volume (cc) of the isodose structures generated at specific dose levels with the SD as the systematic error for the means and the SME for the mean difference. The level for statistical significance was set to *p* < 0.05

Isodose Level	V25.0 Gy	V37.5 Gy	41.7 Gy	V45.8 Gy	V54.2 Gy
	Mean ± SD (95% CI)	Mean ± SD (95% CI)	Mean ± SD (95% CI)	Mean ± SD (95% CI)	Mean ± SD (95% CI)
Empty Protocol	1048.6 ± 341.2 (888.9–1208.3)	529.0 ± 172.5 (448.3–609.8)	454.0 ± 148.4 (384.6–523.4)	395.4 ± 130.8 (334.2–456.66)	224.9 ± 118.0 (169.7–280.1)
Filling Protocol	1451.2 ± 645.2 (72.2–733.1)	745.1 ± 346.8 (40.8–391.4)	640.3 ± 301.5 (34.2–388.4)	558.3 ± 267.3 (28.2–297.6)	303.4 ± 220.5 (-34.7–191.7)
Mean Difference	402.7 ± 163.2	216.1 ± 86.6	186.3 ± 75.1	162.9 ± 66.5	78.5 ± 55.9
p-value	0.02	0.02	0.02	0.02	0.17

(a)

## Discussion

Flexibility of the oART approach has enabled it to be successfully implemented clinically for MIBC. Real-time oART may be considered the gold standard solution to daily online adaption for MIBC and has been successfully achieved using, for example, MRI-guided linear accelerators and the Ethos™ treatment platform [29, 30]. Meanwhile, proactive oART is still a technically feasible solution which can be readily adopted with clinically effective results [14, 16, 24, 31, 32].

Central to the successful implementation of proactive oART is plan selection. Several authors have highlighted the issue of inter-observer variability in plan selection when employing the plan of the day delivery approach [14–16, 20, 32–34]. Education and training of therapeutic radiographers to overcome inter-observer variability in plan selection is paramount. At our centre, fifteen therapeutic radiographers had previously been QA approved to select the plan of the day for the RAIDER clinical trial. As mentioned earlier a training package was developed to continue to train and assess competency for other therapeutic radiographers outside of the trial. The training package includes using a Varian testing station also known as a training box (T-box) for offline training and testing of plan selection. Final assessment is carried out by an IGRT advanced therapeutic radiographer and is undertaken after the trainee has completed a training log sheet of online plan of the day selection under the supervision of two competent therapeutic radiographers. Ultimately, a robust training programme should be considered when implementing a plan library approach as this can ensure a high concordance of plan selection for therapeutic radiographers [14, 18].

This study has demonstrated significant differences in bladder (CTV) volumes and PTV volumes when comparing a BE protocol to a BF protocol. Dees-Ribbers et al. [35] compared empty and full bladders in their study of inter- and intra-fractional bladder motion during radiotherapy and reported median bladder volumes of 136 ml and 213 ml for empty and full bladder protocols, respectively. McDonald et al. [16] using an empty bladder protocol recorded a mean bladder volume of 140 cc on CT0, 159 cc on CT30 and 113 cc from on-treatment CBCTs. In this work we found comparable mean bladder volumes of 131.6 ± 19.3 cc on CT0, 171.9 ± 21.6 cc on CT30 and 117.3 ± 14.8 cc on pre-treatment CBCTs with larger mean bladder volumes being recorded for the BF protocol 231.4 ± 29.9 cc (CT30), 314 ± 35.5 cc (CT60) and 191.2 ± 23.3 cc (CBCT). Bladder volumes used for treatment planning are therefore different in both protocols with the BE protocol illustrating consistently and statistically smaller bladder volumes (*p* < 0.05).

As illustrated in Fig. 2, mean bladder volumes on treatment were reduced compared to pCT volumes, which is consistent with previously published literature [7, 9, 16, 36]. Foroudi et al. [10], observed a decrease in bladder volume with fraction number due to more frequent bladder voiding resulting from treatment-induced irritation. Bladder volumes were also shown to decrease during treatment for both protocols examined here with a significant difference being recorded between the first and final fractions of treatment (Fig. 3). As part of our retrospective audit, bladder filling rates were assessed on pCTs only, however, our results fall within a range of previously published values, 0.9–4.0 ml/min [35].

Similarly, studies pertaining to IGRT for prostate cancer have also indicated that treating with an empty bladder may improve consistency and that larger bladders at the time of CT acquisition is indicative of a larger variability during treatment [37, 38]. Our data suggests that bladder volume reproducibility is more readily achieved using a BE protocol.

For the analysis of on-treatment plan selection in the BE protocol group our results illustrate that the majority of plans selected during treatment were medium sized (58.6%). Previous studies have found medium sized plans to be deemed most suitable for the majority of patients undergoing oART for bladder cancer [32, 39, 40]. While 47% of plans chosen in the BF protocol group were small, small and medium plans combined accounted for 84.6% and 77.5% of the plans selected within the BE and BF groups, respectively. The choice of small plans on treatment for the BF protocol could be explained by mean bladder volume differences recorded between the pCTs compared to on-treatment CBCTs. While there was no significant difference in volumes for the BE group (Fig. 2), mean pCT volumes (231.4 ± 29.9 cc)were significantly larger than mean CBCT volumes (191.2 ± 23.3 cc) in theBF group (Fig. 2). The selection of mostly small plans for the BF group could therefore be ascribed to bladder volume on treatment being significantly smaller than the volume at simulation. Furthermore, the mean PTV volumes for the medium plans in the BE group and small plans in the BF group are statistically equivalent. Previous studies have indicated similar PTV volumes recorded using a comparable BE protocol (Fig. 6) [16, 17, 19, 41]. Overall, our BE protocol appears to offer more consistent and reproducible bladder volumes at the time of simulation and treatment, with the mean volume of the medium PTV being statistically equivalent to the mean “treated” PTV volume.

As previously mentioned, mean “treated” PTV volumes were significantly greater for the BF protocol group compared to the BE group which in turn resulted in consistently higher doses predicted to have been delivered to the bowel loops and rectum in this group (Tables 2 and 3). With no change to PTV coverage, analysis of the isodose volumes for all patients show that 25.0 Gy, 37.5 Gy, 41.7 Gy, and 45.8 Gy are statistically smaller for the BE protocol (*p* < 0.05). Therefore, we can state with reasonable confidence that patients are receiving less dose with the BE protocol due to the reduction of the target size.

Limitations of this study include its retrospective nature and sample size. In addition, the dosimetric analysis of PTV coverage and OAR doses was based upon structures delineated on the planning CTs. While several studies have explored dose calculation techniques on CBCTs and synthetic CTs [42, 43] this was not currently feasible at our centre. We, therefore, assessed our plans within the boundaries of our clinical workflow. For improved accuracy it would be necessary to re-outline the OARs on the CBCT images and perform the dose calculations on these images. However, the analysis is strengthened by the assessment of the isodose volumes at relevant dose levels which should be insensitive to anatomical changes on treatment.

The CTV to PTV margins as defined in Table 1 for medium sized plans is in close agreement with other anisotropic PTV expansions previously reported in the literature [16, 39, 44]. While optimal CTV to PTV margins for oART for MIBC are still under investigation this lies outside the scope of this paper. However, in light of the results herein we do advocate a BE protocol and support the RAIDER clinical trial CTV to PTV margins in particular those that generate small and medium plans. The use of large PTV plans, which irradiate larger volumes of normal tissue, may be further reduced by ensuring the BE protocol is consistently implemented at the time of CT acquisition and prior to the delivery of each treatment fraction. In clinical reality, bladder filling is not the sole contributor to the geometric uncertainties associated with radical RT for MIBC and bowel preparation protocols could also be considered. All patients received at least two out of their three library plans over the course of treatment and there were no instances when any of the three library plans were deemed clinically unacceptable, confirming that an oART treatment approach is technically and clinically feasible.

## Conclusion

To the best of our knowledge this is the first oART bladder study comparing empty and filling drinking protocols using the CTV to PTV margins from the RAIDER clinical trial. This study has provided compelling evidence supporting the use of a BE protocol in patients receiving oART for MIBC. This protocol shows better reproducibility of CTV volumes at the time of CT acquisition and during treatment, reduced PTV volumes and adequate PTV coverage along with lower OAR doses. Also, a decrease in normal tissue irradiation has also been illustrated which could potentially reduce RT-related toxicities in patients. Overall, these results provide evidence towards the potential benefit of clinically implementing oART with a BE protocol for MIBC.

## Data Availability

No datasets were generated or analysed during the current study.

## Abbreviations

MIBC: Muscle-invasive bladder cancer
PoD: Plan of the day
BE: Bladder empty
BF: Bladder full
pCT: planning CT
PTV: Planning Target Volume
OARs: Organs at risk
VMAT: Volumetric arc therapy
ART: Adaptive radiotherapy
oART: online adaptive radiotherapy
IGRT: Image Guided Radiotherapy
CBCT: Cone Beam Computed Tomography
TPS: Treatment Planning System
